# Systemic nickel allergy syndrome. Biological monitoring of dietary nickel intake and induction of immunotolerance

**DOI:** 10.1186/2045-7022-1-S1-P108

**Published:** 2011-08-12

**Authors:** Cirla Angelo Mario

**Affiliations:** 1 Centro Italiano Medicina Ambiente Lavoro, Section of Allergy, Cremona, Italy

## 

Nickel sensitized patients may suffer of contact dermatitis,but also of urticaria-like, pruritus-erythema and cutaneous rush,sometimes associated with intestinal symptoms.The role of Nickel absorption due to food is still debated, but a clinical framework of Systemic Nickel Allergy Syndrome (SNAS) may be proposed, while a possible induction of oral tolerance deserves to be investigated.

In the same Allergy Unit 152 subjects (126 F,26 M) were diagnosed as allergic by positive patch test to Nickel sulphate (class 2-3). All underwent Urinary Nickel determinations (NiU), by AAS with Zeman corrector and results standardized to creatinine concentration. According to single diagnosis patients were selected. Group A (65 pt) with only contact dermatitis. Group B (87 pt) with skin troubles of SNAS.

At the first determinationt NiU values in Group B (mean 2,25 mcg/g creatinine) were significantly higher when compared to Group A (mean 0,87 mcg/g creatinine), with p<0,0001. Abnormal values measured at free diet exceeded the fixed limit of attention of 1,7 in 55% of Group B (48/87 cases) and only 20% in Group A (13/65). The 48 subjects of Group B received a Nickel-scanty diet that observed for six months; end-NiU resulted below the adopted limit for all patients. In spite of this dietary restriction 30 of them claimed again episodic cutaneous urticaria-like symptoms and frequent itching. 12 subjects were included in an experimental oral treatment (Lofarma Laboratories, Milan) based on subsequent microdoses of Nickel sulphate,from 0,1 to 1000 nanograms (1 mcg), aimed to induce an immunotolerance. Treatments were monitored by NiU. All these cases reported improvement; absence of symptoms was documented after 8 months of maintenance with the highest dose.

SNAS is statistically related to an increased Nickel intake from dietary habit. NiU seems to be a reliable indicator in biological monitoring. A "Nickel-scanty"diet may reduce the incidence of SNAS, according to a decrease of NiU. A surveilled oral treatment by microdoses of Nickel sulphate seems to induce a specific immunotolerance and clinically to realise reduction or suppression of symptoms.

